# OTME-18. Targeted CRISPR/Cas9 gene-editing regulates the brain tumor environment

**DOI:** 10.1093/noajnl/vdab070.069

**Published:** 2021-07-05

**Authors:** Joshua Perez, Javier Fierro, Rocio Aguilar, Huanyu Dou

**Affiliations:** Texas Tech University Health Sciences Center El Paso, El Paso, TX, USA

## Abstract

Glioblastoma multiform (GBM) is the most common malignant brain tumor. Recent immunotherapy has demonstrated potential to treat GBM. However, the immune suppressive tumor environment in the brain represents a significant barrier for the treatment of GBM. Overexpression of programmed death ligand-1 (PD-L1) in GBM tumor cells and macrophages plays a key role in GBM vitality, proliferation, and migration, while also suppressing the immune system. We developed a CRISPR/Cas9 gene-editing system to delete whole cell PD-L1. Human PD-L1 targeted sgRNA were cloned into CRISPR/Cas9 plasmids with or without an HDR templet. CRISPR/Cas9 were treated to human GBM U87 cells for 15, 30, 60, 120 and 240 minutes. The intracellular concentration of CRISPR/Cas9 exhibited a time-dependent increases. A GFP tagged CRISPR/Cas9 plasmid was developed to test the transfection efficacy. Higher levels of GFP+ U87 cells were observed at day 3. CRISPR/Cas9 showed a greater PD-L1 knockout at day 3. The PD-L1 reduction limited the proliferation of U87 cells. A scratch assay showed that PD-L1 deletion inhibited the migration of U87 cells. An in vitro GBM model was developed by co-cultivation of U87 cells and macrophages. CRISPR/Cas9 treated co-cultures changed the ratios of U87 cells and macrophages and polarized tumor associated macrophages (TAM) from M2 toward M1. CRISPR/Cas9 gene-editing effectively deleted PD-L1 in U87 cells. Successful deletion of PD-L1 prevented U87 cells growth and migration, and altered the TAMs plasticity and the tumor environment.

